# Impact of squid predation on juvenile fish survival

**DOI:** 10.1038/s41598-022-14389-2

**Published:** 2022-07-11

**Authors:** Motomitsu Takahashi, Tatsuya Sakamoto, Chiyuki Sassa, Mari Yoda

**Affiliations:** 1 Fisheries Resources Institute, Japan Fisheries Research and Education Agency, 1551-8 Taira-machi, Nagasaki, Nagasaki 851-2213 Japan; 2 Present Address: Instituto Português do Mar e da Atmosfera (IPMA), Rua Alfredo Magalhães Ramalho, 6, 1495-006 Lisbon, Portugal

**Keywords:** Ecology, Behavioural ecology, Biooceanography

## Abstract

Predation is a major source of mortality during the early life stages of marine fishes; however, few studies have demonstrated its impact—especially that of squid predation—on survival processes. Here, we examined the feeding habits and predation impacts of swordtip squid on a major prey fish, juveniles of jack mackerel, in the East China Sea. Otoliths of the juveniles extracted from the squid stomach were used to reconstruct the age–length relationship and the growth trajectory of the consumed juveniles, and they were compared to those of juveniles collected with a net using a newly developed statistical framework. The juveniles consumed by squid had significantly shorter body lengths and smaller body sizes during the late larval and early juvenile stages than the netted juveniles, suggesting that smaller juveniles with slower growth rates have a higher probability to be selected. The body mass ratio of the predator squid to prey juveniles (predator–prey mass ratio, PPMR) ranged from 7 to 700, which was remarkably lower than the PPMR reported in various marine ecosystems based on analyses of fishes. Our findings demonstrate that squid predation can significantly impact the early life survival of fish and the trophodynamics in marine ecosystems.

## Introduction

Predation is a major source of mortality during the early life stages of marine fishes^1^. As population fluctuations in marine fishes are often driven by variabilities in early life mortality rates, assessing the effect of predators on fish survival processes is essential for understanding fish population dynamics. It has been hypothesised that a subtle decline in growth rates during the early life stages results in prolonged vulnerability and increased cumulative mortality due to predation, which potentially results in decreased recruitment^2,3^. Low growth rates may also be related to low standard metabolic rates^4^, which can affect animal activities including predator avoidance^5^. Because predation on marine fish can rarely be directly observed in open oceans, it is necessary to examine phenotypic differences between individual fishes consumed by predators and those surviving in the population to understand the impact of predation on fish survival^6^.

Various marine fish species have a planktonic larval life for dispersal and settle in species-specific habitat layers after metamorphosis for growth and reproduction. This transition from the surface to the deeper layer during early life stages is one of the conditions that select for a fast growth rate in marine fish^7^. The transition often involves dramatic changes in the physical and biological environments, including a new predatory field. Squids often occupy mesopelagic waters and perform significant vertical diel migrations^8,9^. As the reported diet composition of various squids includes numerous fish species^10,11^, squids may play a significant role in the survival process of fishes, especially those that shift their distribution to deeper layers during early life stages. Several studies have reported on the predator–prey relationships between fishes^12,13^ or between seabirds and fishes^14^; however, few have highlighted the relationship between squids and fishes. Although predation by cephalopods has been hypothesised to have a significant impact on the recruitment dynamics of fishes^11,15–17^, there is a critical knowledge gap regarding the impact of squid predation on the survival of fish species.

The swordtip squid *Uroteuthis edulis* and Japanese jack mackerel *Trachurus japonicus*, both abundant in the near-bottom layer (within few meters from the bottom) in the southern East China Sea (ECS)^18–20^, can be a suitable set of species to explore the interactions between squids and fishes as well as the role of squids in marine ecosystems. The Japanese jack mackerel is one of the species that ontogenetically shifts its habitat from the surface to the near-bottom layers when the fish reach approximately 30 to 50 mm in standard length (SL)^19,21^. From the comparison of the growth trajectory between the larvae and early-stage juveniles (~ 20 mm SL) in the surface layer and juvenile conspecifics (30–70 mm SL) in the near-bottom layer of the ECS, Takahashi et al.^22^ showed that *T. japonicus* larvae and early-stage juveniles experience size or growth-selective survival around the habitat shift. In addition, *U. edulis* is reported to prefer small fishes as prey^23,24^. As this species of squid is distributed predominantly in the shelf-break regions during spring and early summer and often co-occurs with *T. japonicus* juveniles, it may be the key predator of this fish species to drive its growth-selective survival.

Behavioural and feeding patterns of squids often vary depending on the time of day^15^ and the lunar cycle^8^, although such patterns of *U. edulis* are yet to be revealed. The weight ratio of the predator and prey referred to as the predator–prey mass ratio (PPMR) is a fundamental parameter that characterise feeding apparatus, controls food chain length in a community of a given size composition^25,26^ and subsequently affects the energy transfer efficiency from phytoplankton to the top predators^27^. Despite its importance, PPMR has rarely been reported in the context of squid predatory behaviour. This is mainly due to the difficulty in weighing consumed prey because squids bite prey into small pieces and digest them rapidly^11,15^. Moreover, cephalopods often reject hard body parts, such as the head and caudal fins, during predation and feed only on the fish trunk^28^. Because of these difficulties, investigating the predator–prey relationship between squid and fish requires careful assessment.

In this study, we aimed to understand the feeding habits of *U. edulis* and its impact on the growth-dependent survival of *T. japonicus* during the fish early life stages in the ECS. For this purpose, *U. edulis* and *T. japonicus* were collected using a bottom trawl in the southern ECS. Using otoliths of *T. japonicus* juveniles found in the stomach of *U. edulis* (hereafter the consumed juveniles) and those collected by a trawl (hereafter the netted juveniles), we were able to reconstruct body sizes and growth trajectories of the juveniles to reveal phenotypic differences between the two groups. To describe feeding habits of *U. edulis*, the PPMR and the digestion state were estimated using the reconstructed sizes of the consumed juveniles. We also developed a statistical framework that utilises the age–body length relationship to determine the size or growth selectivity of fish by squid predation.

## Materials and methods

### Field collection and stomach content analysis

**Figure 1:**
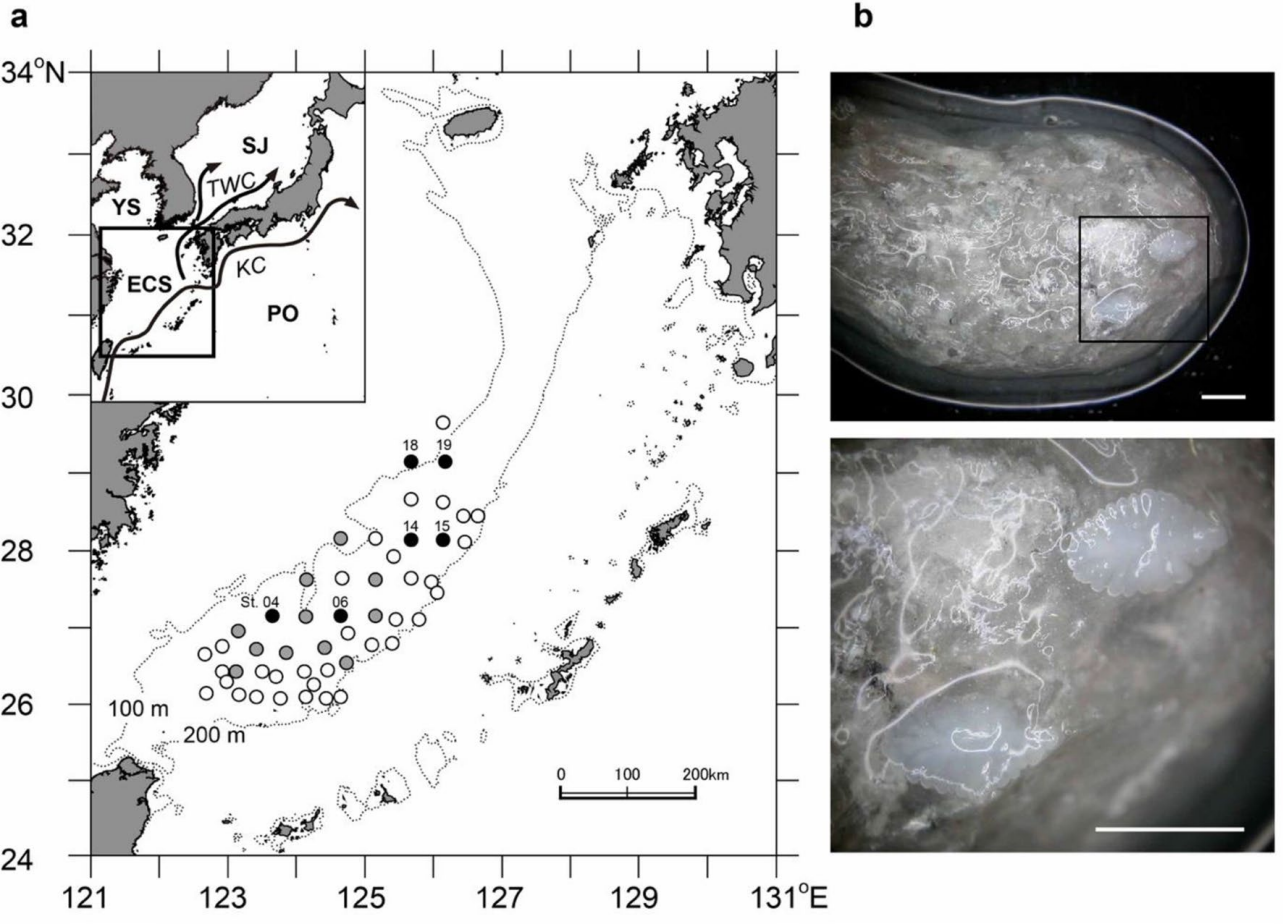
Collection sites of juvenile and adult *Uroteuthis edulis* for stomach content analysis in 2008, 2009, and 2010 in the southern ECS and a stomach of *U. edulis*. (**a**) Collection sites with solid, shaded, and open circles indicate sites with otolith analysis of ≥ 3 consumed *Trachurus japonicus* juveniles, sites with ≤ 2 consumed juveniles, and sites without consumed juveniles, respectively. Location and numbering of trawl stations were consistent among the study years. Inset has the water and major current structures labeled: ECS, East China Sea; YS, Yellow Sea; SJ, Sea of Japan; PO, Pacific Ocean; KC, Kuroshio Current; TWC, Tsushima Warm Current around the study area. (**b**) Stomach contents of *U. edulis* including a pair of saccular otoliths of juvenile *T. japonicus*. The bottom image is an enlarged view of the square in the top image. Scale bars 2 mm.

Juvenile *T. japonicus*, as well as juvenile and adult *U. edulis*, were collected using a bottom trawl from May to June in 2008, 2009, and 2010 in the ECS (Fig. 1a, Table 1). As growth-selective survival in *T. japonicus* juveniles has been observed in the shelf-break region of the ECS south of 30° N in our previous study^22^, we focused on analysing the stomach contents of *U. edulis* in the southern ECS, south of 30° N. The number of trawl stations for stomach content analysis was 30, 39, and 39 in 2008, 2009, and 2010, respectively. Of these trawl stations, those with more than three consumed juvenile individuals were selected as study sites to examine the feeding habits of *U. edulis* and size-selective predation on *T. japonicus* juveniles. Our procedure for collecting fish and squid samples is a fishery survey for assessing recruitment abundance of fish and cephalopods including *T. japonicus* and *U. edulis* in the ECS. The Fisheries Agency of Japan provided approval for sample collection every year. Both species are stock assessment species and the stocks are managed based on stock assessment regulations in Japan.

**Table 1:** Number of individuals of *Uroteuthis edulis* used for stomach content analysis and those of *Trachurus japonicus* used for growth comparison between the consumed juveniles (CJ) and the netted juveniles (NJ) with proportions of the unpalatable individuals in the netted juveniles (*α*_*n*_) in the East China Sea.

Year	Site	Depth (m)	Bottom temperature (°C)	Date	Time	Moon age (Lunar illumination, %)	Number of *U. edulis* analysed stomach		Number of *T. japonicus* analysed growth		
							Total	With CJ (%)	CJ	NJ	*α*_*n*_ (%)
2008	St. 04	114	19.3	21-May	6:29	15.6 (94.2)	50	4 (8.0)	12	46	0.0
2009	St. 06	104	19.2	27-May	6:53	2.6 (17.6)	10	5 (50.0)	5	43	25.6
	St. 15	120	17.8	19-May	6:24	24.0 (37.3)	30	3 (10.0)	7	46	0.0
	St. 18	95	17.7	18-May	9:29	23.0 (44.1)	25	3 (12.0)	3	44	100.0
	St. 19	98	17.8	18-May	6:24	23.0 (44.1)	23	6 (26.1)	10	48	4.2
2010	St. 14	114	18.1	17-May	9:18	3.1 (21.0)	10	3 (30.0)	7	40	2.5
	St. 19	101	18.6	16-May	6:17	2.1 (14.2)	19	10 (47.1)	10	43	74.4

A trawl net was towed at the sea bottom for 30 min during the daytime between 0600 and 1800 local time. The net had a mouth opening of approximately 22 m × 4 m (width × height) and various mesh sizes (180 mm at the mouth to 66 mm at the cod-end) covered with an 18 mm mesh size web liner outside the cod-end. Up to 50 individuals of *T. japonicus* and *U. edulis* were removed from each trawl catch and immediately frozen onboard at − 10 °C. Vertical temperature profiles at the trawl stations were obtained from observations using the salinity-temperature-depth profiler (ASTD-100, JFE Advantech Co. Ltd., Hyogo, Japan).

Mantle length (ML) and wet bodyweight of *U. edulis* were measured with 0.1 cm and 0.1 g accuracy, respectively. Stomachs of *U. edulis* were dissected, and the wet weight was measured with 1 mg accuracy, except for those collected in 2008. Diet composition included crustaceans, cephalopods, and fishes based on the identification of beak, exoskeleton, eyeball, otolith, statolith, and vertebral column morphology, as observed under a dissecting light microscope at 5–20 × magnification. Fish otoliths were removed from the stomach contents identified as fishes, and the total number of otoliths was counted under a dissecting microscope at 10–40 × magnification. Little serious damage to otoliths due to mastication and digestion in the stomach was observed (Fig. 1b). All procedures for fish and squid were conducted in compliance with the “*Guidelines for the Care and Welfare of Cephalopods in Research*”^29^, with the “*Guidelines for handling live fish at FRI*” of the Fisheries Research Institute, Japan Fisheries and Education Agency (FRI), and with recommendations of the ARRIVE Guideline^30^.

### Reconstruction of body size/mass and growth trajectory of *T. japonicus*

Otoliths in the sacculus of *T. japonicus* (known as sagittae; hereafter termed as “saccular otolith”) were separated from total otoliths, including other fish species, in the squid stomach and assigned as left and right based on their morphology. All saccular otoliths of the consumed juveniles were embedded on a glass slide with enamel resin, ground using 2000 grit sandpaper, and polished using 3 μm alumina powder. The otolith radius from the core to the margin, the total number of growth increments, and width between increments were measured in the post rostrum portion of the otolith using an otolith measurement system consisting of a light microscope at 50–500 × magnification equipped with a CCD camera (Ratoc System Engineering Co. Ltd., Tokyo, Japan).

SL and wet bodyweight of the consumed juveniles were estimated based on relationships between otolith radius and SL and between wet weight and SL of *T. japonicus* larvae and juveniles (Supplementary Information S1; Fig. S1). As otolith measurements of planktonic larvae and early-stage juveniles of *T. japonicus* in the surface layer and juveniles in the near-bottom layer have already been conducted for 2005, 2008, and 2009 year classes in the ECS in our previous study^22^, the previous data sets of otolith and body sizes in the southern ECS were used in addition to the new measurements conducted for the 2010 year class. When multiple otolith pairs (left and right) were found in the stomach of *U. edulis*, a pair of *T. japonicus* otoliths with differences in estimated SL < 2 mm were defined as originating from the same individual fish. The left-side otoliths were used to reconstruct the SL of the consumed juveniles.

In each study site, SL and wet weight of the netted juveniles of *T. japonicus* were measured with 0.01 mm and 0.1 g accuracy, respectively, and saccular otoliths of the netted juveniles were extracted from up to 50 individuals. The saccular otoliths of the netted juveniles were treated as described for the consumed juveniles. Standard length at ages of the consumed and netted juveniles was back-calculated using the biological intercept method^31^. For *T. japonicus*, growth increments were deposited daily in a saccular otolith and the first growth increment deposition in the otolith was observed 2 days post-hatch (dph) at approximately 3 mm in notochord length in rearing experiments^32^. Age (in days) of the consumed and netted juveniles was estimated by adding 2 to the total number of otolith increments. As the SL–otolith radius relationship was represented by a linear equation (Supplementary Information S1; Fig. S1), parameters of the linear relationship were approximated using SL and otolith radius at hatch and collection for each fish.

### Analysis of *U. edulis* feeding habits

To estimate the time at predation, the digestion ratio (DR) of individual *U. edulis* was estimated by dividing the wet weight of the stomach by the reconstructed wet weight of juvenile *T. japonicus* into cases where *T. japonicus* was only present. As the water temperature at the near-bottom layer of the study sites ranged from 17 to 19 °C (Table 1), the digestion duration (*t*, in min) was estimated based on a linear equation of the gastric evacuation rate of Japanese common squid *Todarodes pacificus* [*DR* = − 0.176 *t* + 59.462 (*N* = 15, *R*^2^ = 0.4101)] at 17°C^33^. Time at predation was estimated by subtracting the digestion duration from the time of catching *U. edulis*, which was defined as the midpoint of trawl towing.

The occurrence rate of the consumed *T. japonicus* juveniles was defined and calculated as the proportion of individual *U. edulis* consuming *T. japonicus* to the total number of analysed *U. edulis* stomachs at each study site. The occurrence rate of the consumed juveniles and time at predation were analysed in relation to the moon stage at the date of predation, which was retrieved from a moon stage tracking website (http://koyomi.vis.ne.jp/moonage.htm). Since the lunar illumination cycles are approximately 29.5 days, the moon stage was standardised based on the proportion of illumination from 0% (new moon) to 100% (full moon). To elucidate the prey size preference of *U. edulis* and its association with ontogeny and lunar cycles, the total mass of *T. japonicus* juveniles consumed by *U. edulis* and the PPMR were calculated as the wet weight of *U. edulis* divided by the reconstructed wet weight of individual *T. japonicus*, and then compared to the ML of *U. edulis* and lunar illumination using regression analyses.

### Analysis of size/growth-selective predation on *T. japonicus*

Because squids often selectively reject the head of preyed fish depending on their relative body size, the possibility of rejection was investigated before testing the selectivity of squid predation. In a captive study of the European squid *Loligo vulgaris* that were fed fish of approximately 12 g in wet weight, selective rejection was mainly observed in squids approximately < 19 cm ML with a probability of 60–100%^28^, suggesting that the head of fish larger than 1/13.2 of the squid in body mass may often be rejected^34^. We, therefore, assumed that selective rejection by *U. edulis* occurs for *T. japonicus* at PPMR < 13.2. The wet weight of *U. edulis* was divided by 13.2 and converted into SL using the wet weight–SL relationship of *T. japonicus* (Supplementary Information S1; Fig. S1), and then averaged at each study site. The mean SL of *T. japonicus* corresponding to 13.2 PPMR was defined as the threshold SL where significant prey head rejection may occur. Hereafter, we refer to the individuals whose SL has exceeded this threshold as the “unpalatable individuals”. For the population whose proportion of the unpalatable individuals is below 5%, even the 100% rejection of the unpalatable individuals will have a limited impact on resulting SL composition if normally distributed as the mean decreases by less than 0.1 standard deviation. If the rejection has indeed occurred, the otoliths of the unpalatable individuals will be found in a reduced proportion in the stomachs of squids. If a squid randomly predated on a fish population that includes *α* % unpalatable individuals and the probability of head rejection for them was 60–100%^28^, the expected proportion of the unpalatable individuals in the stomach will be 0 to 0.4 × *α* × 100/(100–0.6*α) %. Thus, when the proportion of the unpalatable individuals in the netted juveniles (*α*_*n*_ %) was larger than 5%, and the proportion in the consumed juveniles was smaller than 0.4 × *α*_*n*_ × 100/(100–0.6*α_*n*_) %, we considered that significant head rejection may have occurred at the study site and excluded the site from some analyses described below.

**Figure 2:**
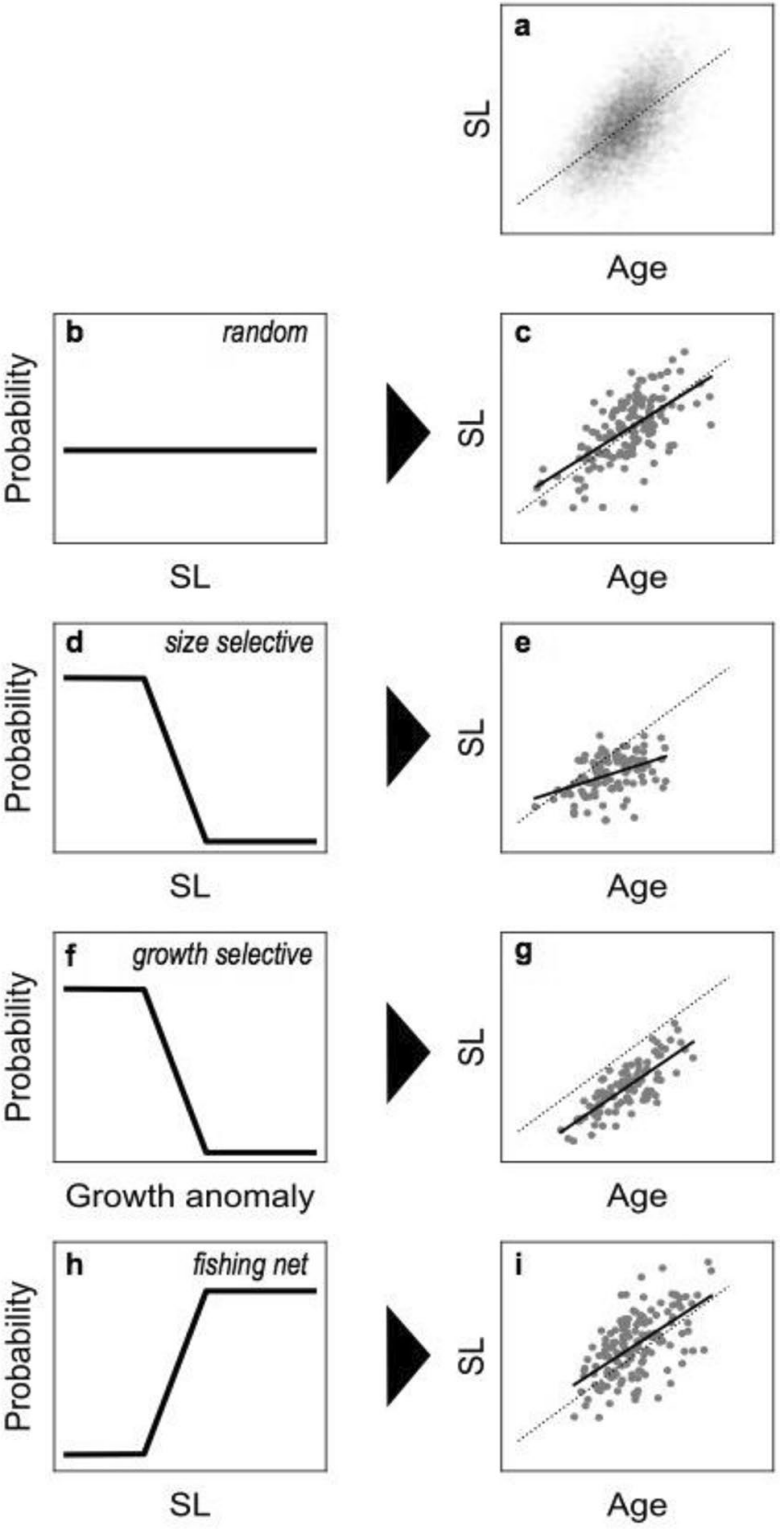
Conceptual models of the effect of predation and net sampling on age–standard length (SL) relationships of consumed or collected specimens. (**a**) Age-SL relationship of the original population consisted of 10,000 individuals with the mean relationship indicated as a dotted line. (**b**–**g**) Schematics of predation probability of random predation (**b**), size-selective predation (**d**), and growth-selective predation (**f**), and the resulting age-SL relationships of consumed juveniles for each predation model with linear regression (solid line) (**c**,**e**,**g**). (**h**,**i**) Schematic of the collection probability of the fishing net (**h**) and its resulting age-SL relationship of collected juveniles (**i**).

**Figure 3:**
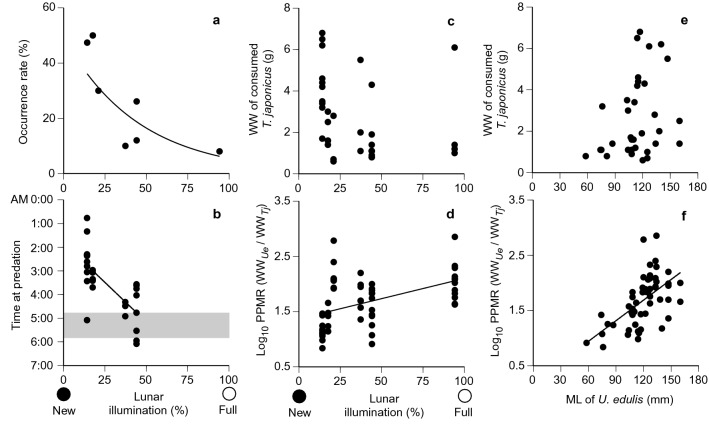
Predation of *Trachurus japonicus* juveniles in relation to lunar illumination. (**a**) Occurrence rate and (**b**) time at predation. Shaded area in (**b**) indicates twilight time approximately one hour before sunrise. (**c**–**f**) Wet weight of the consumed *T. japonicus* juveniles (**c**,**e**) and predator–prey mass ratio (PPMR) (**d**,**f**) in relation to lunar illumination and *Uroteuthis edulis* mantle length (ML). Solid lines indicate significant relationships between variables.

The age-SL relationships of the netted and consumed juveniles were used to test the size or growth selectivity of squid predation (Fig. 2a). Here, three models of predation selectivity were considered: (1) random predation (no selectivity, Fig. 2b), (2) size selection (small size preferred, Fig. 2d), and (3) growth selection (slow growth rate preferred, Fig. 2f). A small size refers to a relatively shorter SL regardless of age in a population. A slow growth rate is defined as a relatively shorter age-standardised SL, which can indicate poor nutritional conditions^35^ or low standard metabolic rates^4^, which may subsequently result in the deterioration of swimming ability and an increase in predation vulnerability within a cohort^36,37^. As the three models would result in a different age-SL relationship of the consumed juveniles (Fig. 2c,e,g), the most likely model of predation selectivity can be inferred by comparing the age-SL relationships of the original population and consumed juveniles (Supplementary Information S2 for detailed descriptions). As sizes of the consumed juveniles skewed to smaller ranges of that of the netted juveniles (see “Results”), we tested the size selection only for smaller sizes. Notably, fishing nets can fail to capture individuals smaller than their mesh size (Fig. 2h), and thus the age-SL relationship of the netted juveniles does not directly represent that of the original population and may have a higher mean length and shallower slope (Fig. 2i).

Based on the abovementioned concept, a statistical framework to infer the likely predation model was developed (see Supplementary Information S2 for detailed descriptions). Briefly, the age-SL relationship of the original population, defined as the population prior to predation and net sampling, was first inferred from the age-SL relationship of netted juveniles using weighted linear regression analysis to account for net bias. Next, the model of predation on the original population that can best explain the observed age-SL relationship of consumed juveniles was selected based on Akaike’s Information Criteria (AIC) and bootstrap likelihood ratio tests with 2,000 iterations using the random predation model as the null hypothesis. These procedures were applied to the data for each study site, pooled data for each year, and pooled data for all years to cover various spatiotemporal scales^38^, although study sites where significant selective rejection for feeding may have occurred were excluded. Importantly, when size selection occurred in multiple populations of different age ranges and the data were pooled, the age–SL relationship of the pooled consumed juveniles will be similar to that under growth selection. Therefore, although size and growth selectivity are different feeding strategies, both models are essentially indistinguishable and may differ only at the local level.

Size- or growth-selective predation was detected in most study sites or years (see “Results”). To further elucidate the phenotypic differences in somatic growth trajectories between netted and consumed juveniles, back-calculated SL at different ages was compared in each year. As the age-SL relationships showed bi- or tri-modal distribution in each year (see “Results”), the netted and consumed juveniles were assigned using a cluster analysis based on SL and age. Clustering using Ward’s method was implemented to construct the dissimilarity matrices. Differences in growth trajectories at 5-day intervals between the consumed and netted juveniles were tested using repeated measures MANOVA with a post hoc F-test^39^. Statistical analyses were conducted using JMP version 14.0 software (SAS Institute, Cary, NC, USA; www.sas.com).

### Ethics declaration

Ethical review and approval were not required for this study because the provision of animal welfare of the FRI did not require researchers to submit protocols for the ethical treatment of fish and invertebrate collected in the field survey when this research was conducted.

## Results

### Abundance and biomass of fish and squid species in the study area

Among the various species found during the surveys in the southern ECS, *U. edulis* had the highest mean abundance in all survey years, and *T. japonicus* had the second-highest mean abundance in 2008 and 2009, but was sixth in 2010 (Supplementary Information S3; Fig. S2). The occurrence of *T. japonicus* ranged from 64 to 82% in the trawl stations during the study period (Supplementary Information S3; Fig. S2). *U. edulis* was captured in 100% of the stations in which *T. japonicus* juveniles were found, which was the highest percentage among all fish and squids found in the surveys. For other potential squid predators, multiple species of cuttlefish and the Japanese common squid *T. pacificus* co-occurred with *T. japonicus*, although the mean abundance of these squids was one to two orders of magnitude lower than that of *U. edulis*. A similar predominance of *U. edulis* and *T. japonicus* was found in the mean biomass analysis (Supplementary Information S3; Fig. S2).

### Feeding habits of *U. edulis* on *T. japonicus*

Juveniles and adults of *U. edulis* feeding on *T. japonicus* juveniles were collected in the shallow (95–120 m) shelf-break region of the ECS mainly during the early morning; reduced predation occurred in the southern marginal shelf and deeper areas (> 150 m) (Fig. 1a, Table 1). Study sites with more than three individuals of consumed *T. japonicus* juveniles were St. 04 in 2008; St. 06, 15, 18, and 19 in 2009; and St. 14 and 19 in 2010. Occurrence rates of the consumed juveniles were 8–50% in the study sites and were higher during lower lunar illumination close to the new moon period than during higher illumination around the full moon period (Table 1, Fig. 3a).

ML and wet weight of *U. edulis* that consumed *T. japonicus* juveniles ranged from 58 to 160 mm and 9.5 to 122.1 g, respectively (Supplementary Information; Table S1); no significant difference was found in ML and weight among years (ANOVA, *P* > 0.5 for both). A swordtip squid *U. edulis* consumed 1–7 individuals of *T. japonicus* juveniles with an estimated body length of 20.0–74.9 mm SL. Digestion rates of the consumed juveniles were approximately 1–71% at 88–330 min after predation, based on the digestion states (Supplementary Information; Table S1). Predation was estimated to occur between 0100 and 0400 during the lower lunar illumination period and between 0400 and 0600, including twilight time, during the middle illumination period (Fig. 3b); data on predation time around the full moon period is not available because stomach wet weight was not measured in 2008. No significant trend was found between the bodyweight of the consumed juveniles and lunar illumination (*P* = 0.14, Fig. 3c). However, the PPMR during the full moon period was higher than during the new moon (*P* < 0.01, Fig. 3d). While wet weights of the consumed juveniles in *U. edulis* with ML > 100 mm tended to be higher than those consumed by the squid with ML < 100 mm (Fig. 3e), there was no significant relationship between the weight of the prey and predator (*P* = 0.08). However, the PPMR was significantly higher for *U. edulis* with longer MLs (*P* < 0.01, Fig. 3f).

### Size/growth-selective predation

**Figure 4:**
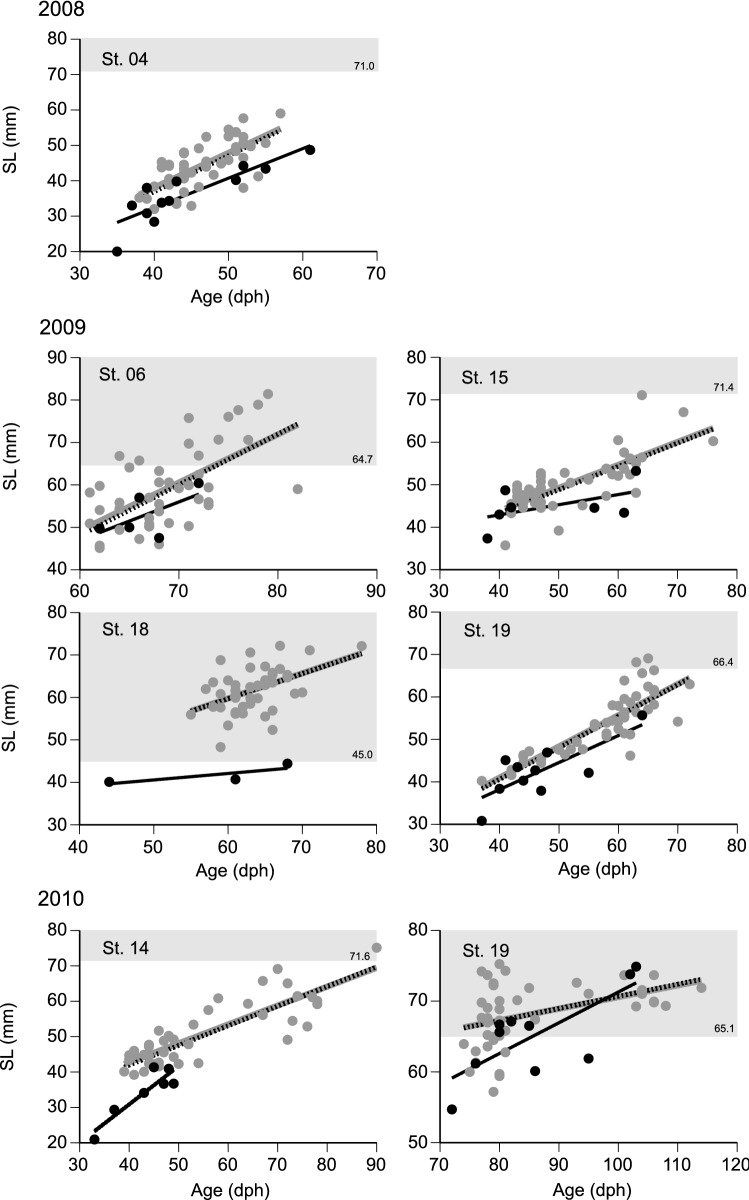
Relationships between standard length (SL) and age of the consumed (black circles) and the netted (grey circles) juveniles of *Trachurus japonicus* in the study sites. The thick solid grey and black lines indicate linear regression of the netted and consumed juveniles, respectively. Dotted black lines indicate the age–SL relationship taking into consideration net bias for the netted juveniles. Shaded area indicates potential SL ranges with the selective rejection of feeding by squids, which corresponds to a predator–prey mass ratio (PPMR) < 13.2. Numbers at the bottom right in the shaded area indicate the threshold SL of the potential selective rejection.

The age-SL relationships of populations prior to net sampling and squid predation (i.e., original population) were estimated using linear regression analyses of the age-SL data of net samples. Weighted regression analysis considering net bias resulted in a slightly different mean age-SL relationship from that estimated by non-weighted regression analysis for most study sites, indicating that the net bias hardly affects the age-SL relationship (Fig. 4). Less than 5% of netted juveniles at each study site had longer SLs than the threshold (PPMR < 13.2, see “Materials and methods”), indicating that selective rejection of the prey head had unlikely to have occurred, except in St. 06 and St. 18 in 2009 and St. 19 in 2010 (Fig. 4, Table 1). At St. 19 in 2010, 60% of the consumed juveniles had longer SLs than the threshold (expected proportion under rejection: 0 to 53.8%, see “Materials and methods”), suggesting that the heads of prey were not rejected despite their relatively large sizes. We assumed that significant selective rejection did not occur except in St. 06 and 18 in 2009, and conducted further analysis excluding these two sites.

**Figure 5:**
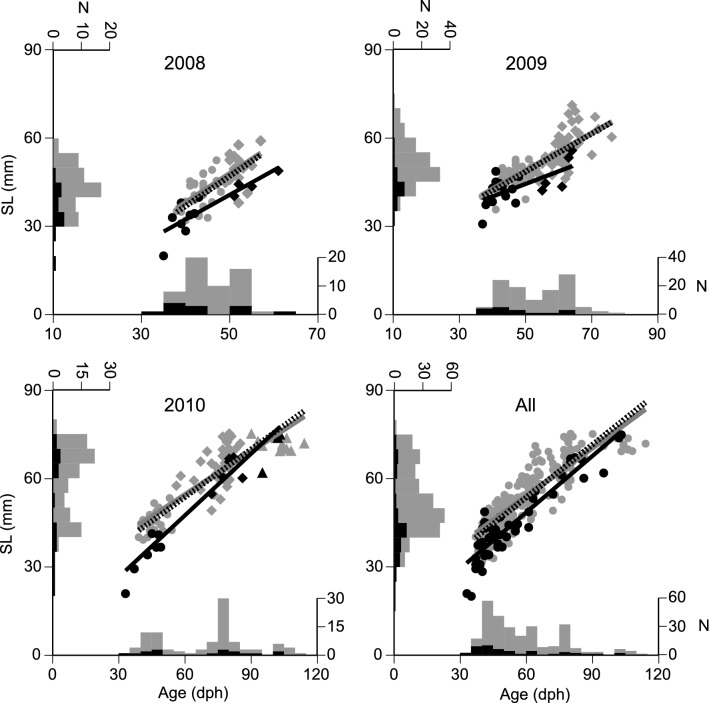
Relationships between standard length (SL) and age of the consumed (black) and netted (grey) juveniles of *Trachurus japonicus* across the study years and pooled years. The thick solid grey and black lines indicate linear regression lines of the netted and consumed juveniles, respectively. Dotted black lines indicate the age-SL relationship taking into consideration net bias for the netted juveniles. Frequency distributions for SL and age in each panel indicate the number of individuals in the 5 mm and 5 dph classes, respectively. Circles and diamonds in 2008 and 2009 indicate younger and older age cohorts based on the cluster analysis. In 2010, circles, diamonds, and triangles show younger, middle and older age cohorts.

In comparisons of age-SL relationships of the consumed juveniles and the original population, either the size or growth-selective model had a significantly higher likelihood than the random predation model at all study sites except for St. 19 in 2010 (*P* < 0.05), and both were selected for AIC-based model selection (Table 2). When the data were pooled for each year, likelihoods of both size and growth selection models were significantly higher than the random predation model across all three years (*P* < 0.05); the size selection model was selected for 2008 and 2009, and the growth selection model for 2010 (Fig. 5, Table 2). When all years were combined, the likelihood of both size and growth selection models was significantly higher than that of the random predation model (*P* < 0.01), and the growth selection model had the lowest AIC (Fig. 5, Table 2).

**Table 2:** Log likelihoods and Akaike’s Information Criteria (AICs) for the models expressing predation selection by *Uroteuthis edulis* on *Trachurus japonicus* juveniles in the East China Sea.

Site/year	No. of consumed juveniles	Log Likelihood			AIC		
		Random	Size selection	Growth selection	Random	Size selection	Growth selection
2008 St. 04	12	− 41.456	− 33.983**	− 34.424**	82.912	**71.967**	72.848
2009 St. 15	7	− 22.832	− 19.843*	− 21.664	45.665	**43.686**	47.327
2009 St. 19	10	− 32.156	− 29.759	− 29.456*	64.311	63.517	**62.913**
2010 St. 14	7	− 40.684	− 28.694**	− 26.295**	81.368	61.388	**56.590**
2010 St. 19	10	− 32.623	− 31.194	− 30.147	65.246	66.389	**64.293**
2008	12	− 41.456	− 33.983**	− 34.424**	82.912	**71.967**	72.848
2009	17	− 58.080	− 52.301**	− 54.063*	116.160	**108.602**	112.127
2010	17	− 74.206	− 70.567*	− 64.110**	148.412	145.134	**132.221**
All	46	− 178.398	− 169.636**	− 160.467**	356.796	343.272	**324.934**

* and ** indicate *p* < 0.05 and *p* < 0.01 in the bootstrap likelihood ratio test against the random predation model. Bold letters in AIC note the minimum AIC among the three models.

**Figure 6:**
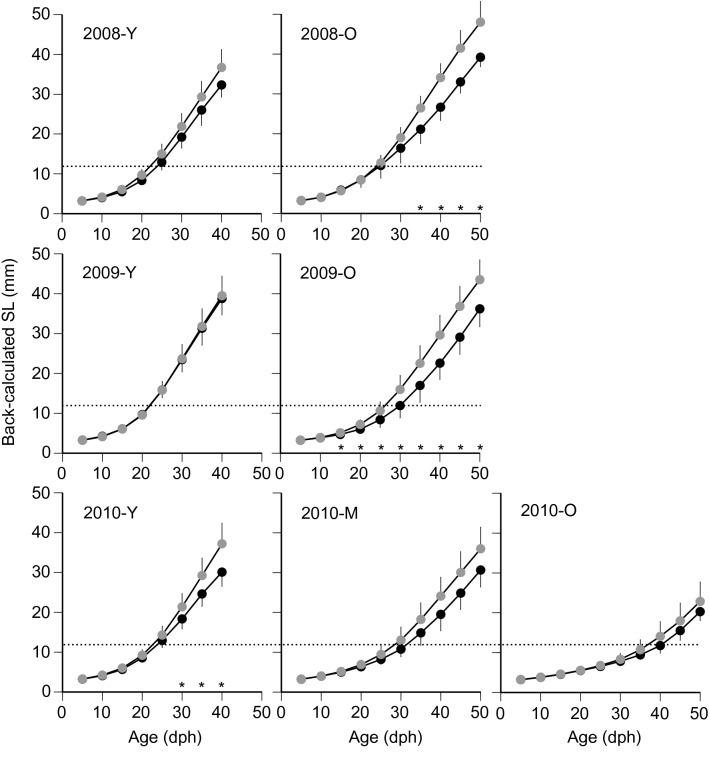
Growth trajectories of the consumed (black) and the netted (grey) juveniles in the young (Y), middle-aged (M), and old (O) groups between 2008 and 2010. Vertical lines indicate the standard deviation of the means. *P < 0.05 in back-calculated standard length (SL) between the consumed and netted juveniles. Horizontal dotted lines indicate SL at *Trachurus japonicus* metamorphosis from larval to juvenile stages^32^.

Using cluster analysis, the consumed and netted juveniles were assigned into two age cohorts in 2008 and 2009 and three cohorts in 2010 based on the age-SL relationships in each year (Fig. 5). Growth trajectories for the younger groups were significantly different between the consumed and netted juveniles only in 2010 (*P* < 0.01), and the mean back-calculated SL of the consumed juveniles was significantly shorter than that of the netted juveniles (Fig. 6). Growth trajectories of the older groups were also significantly smaller than of the netted juveniles in 2008 (*P* < 0.05) and 2009 (*P* < 0.01), whereas no significant difference was found for the middle-aged (*P* = 0.15) and older (*P* = 0.98) groups in 2010. Significant differences in growth trajectories between the consumed and the netted juveniles occurred in the age range of 30–35 dph with back-calculated SL ranging from 20 to 25 mm in 2008 and 2010; in 2009, differences appeared after 15 dph, corresponding to an SL of approximately 5 mm.

## Discussion

In this study, we investigated the predation selectivity of the swordtip squid *U. edulis* on juveniles of the Japanese jack mackerel *T. japonicus* in the ECS during the early summer using otoliths collected with a bottom trawl and those consumed by *U. edulis*. The developed statistical framework detected traces of size or growth-selective predation from the age-SL relationships at most of the analysed study sites and years (Figs. 4, 5, and Table 2). Furthermore, the growth trajectory of the consumed juveniles was consistently smaller than that of the netted juveniles in nearly half of the hatch cohorts (Fig. 6). We, therefore, concluded that *U. edulis* prefers either smaller sized or slower growing *T. japonicus* juveniles in the region and season. Previous bottom trawl surveys suggested that *U. edulis* and *T. japonicus* tend to scatter in the water column during night time and assemble in the near-bottom layer during daytime in the ECS^40^. As the samples in this study were collected during the daytime and the estimated predation times were from midnight to early morning (Supplementary Information; Table S1), selective predation likely occurred not only in the near-bottom layer but also in a wider depth range in the water column.

The selective predation of *U. edulis* may play a key role in the growth-dependent survival and recruitment dynamics of *T. japonicus*. The habitat of *T. japonicus* ontogenetically shifts from the surface to the near-bottom layers in the ECS at approximately 30–50 mm SL^19,22^. Takahashi et al.^22^ demonstrated that larvae and early juveniles of *T. japonicus* with slow growth rates have a low probability of survival up to the juvenile stage. Years of enhanced growth rates of larvae and early juveniles in the surface layer, which were associated with higher prey abundance, have resulted in years of higher juvenile abundance in the near-bottom layer^41–43^. In addition to *U. edulis*, various predators also feed on *T. japonicus* larvae and juveniles in the ECS, such as juvenile yellowtail *Seriola quinqueradiata* associated with surface drifting algae^44^, and demonstrate size/growth-selective predation^12,13^. Our field surveys showed, however, that *U. edulis* is the most abundant fish predator in the near-bottom layer of the ECS and most frequently co-occurred with *T. japonicus* during early summer (Supplementary Information S3; Fig. S2). In addition, the reconstructed body size of juveniles consumed by *U. edulis* was > 20 mm SL and mostly > 40 mm SL (Fig. 5, Supplementary Information; Table S1). This suggests that size- or growth-selective predation by various predators results in the growth-dependent survival of *T. japonicus* throughout the surface to the near-bottom layers and that *U. edulis* is the primary predator during and after the shift from the surface to deeper layers.

The feeding habits of *U. edulis* also provide insights into the survival processes of *T. japonicus* juveniles. The size of the consumed *T. japonicus* did not show a significant correlation with that of *U. edulis*, but the PPMR significantly increased. This indicates that *U. edulis* tends to consume smaller prey relative to its size as it grows, which is a trend commonly observed in various marine predators^26^. Therefore, smaller sized *T. japonicus* is preferred as prey regardless of *U. edulis* size, which further emphasises the importance of rapid growth for *T. japonicus* juveniles to survive. In addition, predation time of *U. edulis* on *T. japonicus* juveniles varied from around midnight to early morning; *T. japonicus* juveniles of larger size relative to *U. edulis* size (lower PPMR) tended to be consumed during the new moon period than during the full moon period (Fig. 4). This indicates that darkness during the new moon allows *U. edulis* to catch larger *T. japonicus* juveniles and that *U. edulis* is capable of finding and hunting fish under low light conditions, perhaps owing to the excellent eyesight of squids^45^. Therefore, while the shift of *T. japonicus* from the surface to the darker bottom layer is related to the seasonal shift of preferred prey items for *T. japonicus* juveniles^46,47^, it may also mean entry into an area advantageous for *U. edulis* predation. Nevertheless, as predation of *U. edulis* on *T. japonicus* was mainly observed in the shallower waters at a depth of 95–120 m (Fig. 1, Table 1), settlements in deeper regions where *U. edulis* is relatively sparse may be important for *T. japonicus* to reduce mortality rates and increase survival rates.

It is noteworthy that the PPMRs between *U. edulis* and *T. japonicus* juveniles (6.9–714.0, median 49.8) were remarkably lower than the ecosystem mean PPMR reported in various regions, which are often approximately 1 × 10^2^ to 10^4^^26,48^. Based on Hunsicker and Essington^16^, Barnes et al.^26^ demonstrated that the mean PPMR of longfin inshore squid *Loligo pealeii* is 97.7 in the western North Atlantic, which is comparable to the ratio obtained in this study. Regionally, Ohshimo et al.^49^ estimated the mean PPMR in the pelagic system of the ECS and the western Sea of Japan as 3–5 × 10^3^ based on nitrogen stable isotope analysis of zooplankton, 18 fish species, and only one squid species. Squids employ their tentacles first to capture prey fish and hold it with their arms, after which they administer a lethal bite behind the head^28,45,50^. This feeding behaviour probably allows squids to consume larger prey relative to their size compared with fish that swallow prey smaller than their mouth/oesophagus diameter; thus, it is likely that the PPMR of squid and fish is generally lower than that between fishes, as has been indicated by previous studies^15,26^. A lower PPMR may allow more members in the predator–prey relationship within a given size range, which leads to longer food chains^25^, reducing the energy transfer efficiency from phytoplankton to the top predator^27^. This highlights the importance of including squids in such analyses to understand marine ecosystems and the need to further accumulate data on squid predation from various regions.

Diet analysis of squids is difficult because of their unique feeding ecology. The selective rejection of the heads of large prey fish can bias the estimation of prey size composition from stomach contents^15^. As the probability of selective rejection for feeding depends on the relative body sizes of predator squid and prey fish, it can critically impact the comparison between the consumed and the net sampled prey fishes. At more than half of the study sites in this study, the body size of *T. japonicus* collected by fishing nets tended to be smaller than the size at which head rejection was reported to occur (Fig. 4). Because we only included study sites at which rejections were unlikely to have occurred in the downstream analyses, our main conclusion overcomes the bias. Nevertheless, at some study sites, the netted juveniles were in the size range where rejection could have occurred, and the size of consumed juveniles was remarkably smaller than that of netted juveniles (Fig. 4, St. 06 and 18 in 2009). This indicates that *U. edulis* may have actually rejected the head of larger *T. japonicus* at the study sites, which can be easily confounded by size-selective predation. As the threshold (PPMR < 13.2) to detect the rejection and the variable gastric evacuation rate depending on temperature were referred from other squid species^28,33^, the feeding habits of *U. edulis* based on the rearing experiments would refine the outcomes in the field. Careful examination of raw stomach contents and size compositions of predators and prey are of critical importance for an accurate understanding of squid predation.

Overall, our results demonstrated that squids could significantly affect the survival process of fish during the transition from the planktonic larval stage to the juvenile stage and settling near the bottom layer, and that the predator–prey relationships between squid and fish may be unique. The transition is widely observed among marine fish species, such as commercially important cods, flatfish, and rockfish. As the variability of survival during early life stages is the essential determinant of population fluctuation of fishes, squid predations have significant impacts on the population dynamics of those fishes. Cephalopod populations have globally increased during the recent decades in various marine ecosystems^51^, suggesting that the predation pressure of squid on fish survival may become increasingly important in future oceans. Although both small fishes and squids are known to be targeted by various predatory fishes^52^ and seabirds^53^, little is known about the interactions between them. Our findings highlight the importance of considering squid ecology to understand fish population dynamics and trophodynamic interactions in marine ecosystems.

## Supplementary Information

Supplementary Information.

## Data Availability

The datasets and the software generated during and/or analysed during the current study are available in the following repository, [10.5061/dryad.7m0cfxpvw] and [10.5281/zenodo.5598679].
