# Supplementary material for: Impact of squid predation on juvenile fish survival
Source: Sci Rep. 2022 Jul 11;12:11777. doi: 10.1038/s41598-022-14389-2 (PMC9276823; doi:10.1038/s41598-022-14389-2)
Supplement: Supplementary file 1 — Supplementary Information. [file 41598_2022_14389_MOESM1_ESM.docx]

**Supplementary Material to the manuscript**

**Impact of squid predation on juvenile fish survival**

Motomitsu Takahashi*, Tatsuya Sakamoto**, Chiyuki Sassa and Mari Yoda.

Fisheries Resources Institute, Japan Fisheries Research and Education Agency, 1551-8 Taira-machi, Nagasaki, Nagasaki 851-2213, Japan.

*Corresponding author: Tel: +81-95-860-1635
Fax: +81-95-850-7767
E-mail: takahamt@fra.affrc.go.jp

**Present Address: Instituto Português do Mar e da Atmosfera (IPMA), Rua Alfredo Magalhães Ramalho, 6, 1495-006 Lisbon, Portugal

**Supplementary Information**

**S1. Relationships between SL and otolith radius and between wet weight and SL**

As interannual variability in the growth trajectory of *Trachurus japonicus* has been reported in the East China Sea (ECS)^S1^, the standard length (SL)–otolith radius (OR) and the wet weight (WW)–SL relationships may differ across the years. Year-to-year differences in the SL–OR and WW–SL relationships were tested using Student’s *t*-test with an analysis of covariance (ANCOVA) with OR and SL as covariates. SL, OR, and WW were natural log-transformed in ANCOVA. The SL–OR relationships were represented using linear equations and were significantly different among the survey years (*P* < 0.001; Fig. S1). The slopes were also significantly different among the years (*P* < 0.001). The SL–OR relationships in 2008, 2009, and 2010 were represented as [*SL* = 0.0461 *OR* + 1.80, *R*^2^ = 0.991, *P* < 0.001, *N* = 226], [*SL* = 0.0482 *OR* + 0.725, *R*^2^ = 0.980, *P* < 0.001, *N* = 323], and [*SL* = 0.0467 *OR* + 0.621, *R*^2^ = 0.974, *P* < 0.001, *N* = 199], respectively, and each equation was applied to reconstruct the SL of the consumed juveniles. The relationships between WW and SL were represented using a power function and were significantly different among the survey years (*P* < 0.001); slopes of the WW–SL relationships were also significantly different among years (*P* < 0.001; Fig. S1). The power function for 2008, 2009, and 2010 were represented as [*WW* = 0.0000162 *SL* ^2.98^, *R*^2^ = 0.997, *P* < 0.001, *N* = 226], [*WW* = 0.00000478 *SL* ^3.27^, *R*^2^ = 0.997, *P* < 0.001, *N* = 323], and [*WW* = 0.00000590 *SL* ^3.22^, *R*^2^ = 0.994, *P* < 0.001, *N* = 199], respectively, and each equation was applied to reconstruct the WW of the consumed juveniles.

Previous studies have reported that damage to the exoskeleton of crustaceans and the skeleton of fishes due to mastication by the squid beak and decalcification by the stomach introduces bias in the reconstruction of prey body size^S2^. In this study, the damage to the otoliths was likely negligible. A few broken otoliths were found, suggesting that otoliths of *T. japonicus* 2–7 cm SL were small enough to be swallowed by *U. edulis* 6–16 cm in ML (Table S1). There was also little damage to the otoliths via decalcification by the stomach acid judging from their surface luster and finely serrated edges (Fig. 1). In rearing experiments of the South African squid *Loligo vulgaris reynaudii*, the food was almost undigested under a decrease in pH from 6.4 to 6.2 during the first 4 h after feeding, which was comparable to the digestion duration estimated in this study^S3^.

**Figure S1**. Standard length (SL)–otolith radius (OR) and wet weight (WW)–SL relationships. Left panel, relationships between SL and OR; right panel, and between WW and SL of *Trachurus japonicus* larvae and juveniles collected from the surface layer in April and those collected from the demersal layer between May and June in 2008–2010. The Y axis for 2009 is shown on the right side of the panels. The bottom panels indicate only linear and allometric regression lines for the SL–OR and WW–SL relationships in each survey year.

**S2. Developing a statistical framework to detect size/growth selective predation**

Three models of predation selectivity were considered: (1) random predation (no selectivity), (2) size selection (small size preferred), and (3) growth selection (slow growth rate preferred). Small size refers to shorter SL regardless of age in a population, and slow growth rate is defined as a shorter age-standardized SL. The three models resulted in different age–SL relationships of the consumed juveniles (Fig. 2). Under random predation (Fig. 2b), the age–SL relationship of the consumed juveniles would not be significantly different from that of the original population (Fig. 2c) because it is a simple subsampling. However, under size selection (Fig. 2d), the consumed juveniles would have shorter mean SL and an age–SL relationship with a shallower slope (Fig. 2e) because younger individuals and older but relatively smaller individuals would be mainly selected. Under growth selection (Fig. 2f), the age–SL relationship of the consumed juveniles would shift lower with a slope similar to or slightly shallower than that of the original population depending on the variance structure of the original population (Fig. 2g); this is because relatively small individuals at each age would be selected across the age range. When size selection occurred in multiple populations of different age ranges and the data were pooled, the age–SL relationship of the pooled consumed juveniles looked like that under growth selection. Thus, although size and growth selectivity can be distinguished from random predation by examining age–SL relationships of the original population and the consumed juveniles, both models are essentially indistinguishable and differ only at the local level.

To develop the framework, we first inferred the age–length relationship of the original population, defined as the population prior to predation and net sampling (Fig. 2a), based on the age–length relationship of netted juveniles. The age–length relationship of the original population was assumed to be linear with a variance structure that increases proportionally with age ($SL \sim a*age+b+N\left( 0,\sqrt{c*age} \right)$). The probability density function of the SL at a given age for the original population (*P_o_*(*SL, age*)) can be written as:

$$P_{o}\left( SL,age \right)=\frac{1}{\sqrt{2\pi}*\sqrt{c*age}}*e^{-\frac{\left( SL-a*age-b \right)^{2}}{2*c*age}} .$$

The diagonal of the mesh inside the bottom trawl net used in this study was approximately 14 mm^S4^, corresponding to the body depth of *T. japonicus* juveniles with approximately 55 mm SL^S5^, although *T. japonicus* as small as 20 mm in folk length (equivalent to 18 mm SL) can still be captured using the net^S4^. This suggests that *T. japonicus* juveniles < 55 mm SL have the possibility of extruding through the mesh, but those > 18 mm SL are retained, with the probability of this increasing with fish size. Because of this probabilistic dropping, the probability density function for the netted juveniles (*P_n_*(*SL*, *age*)) was weighted by a polygonal line function (*w*(*SL*)) and standardized as follows:

$$w_{net}\left( SL \right)=\left\{ \begin{aligned} 0, (SL<18) \\ \frac{(SL-18)}{37}, (18\leq SL\leq55) \\ 1, (SL>55) \end{aligned} \right.$$

$$P_{n}\left( SL,age \right)=\frac{P_{o}\left( SL,age \right)*w_{net}\left( SL \right)}{\int_{-\infty}^{\infty} P_{o}\left( SL,age \right)*w_{net}\left( SL \right) dSL}$$

where *a, b,* and *c* are the parameters in the age–length relationship of the original population. The likelihood *L_n_* was calculated as

$$L_{n}=\prod_{i} P_{s}\left( {SL}_{i}, {age}_{i} \right),$$

where *SL_i_* and *age_i_* are the SL and age of a netted juvenile *i*, respectively, and parameters that maximize *L_n_* were estimated and considered to represent the age–length relationship of the original population. To visualize the effect of the weighting, parameters *a, b,* and *c* were also estimated without the weighting (weighted: dotted black, without weighting: solid grey lines in Fig. 4 and 5).

Next, the likely predation model to obtain the observed age–length relationship of the consumed juveniles was selected. Similar to net sampling, predation can be regarded as a subsampling from the original population and predation selectivity as the probabilistic weighting during subsampling. Probability density functions for the consumed juveniles under each model can therefore be described by weighting and standardizing the fitted probability density function for the original population ($\bar{P_{o}}\left( SL,age \right)$). The weighting functions for each model (*w_random_, w_size selection_*, and *w_growth selection_*) were defined as follows:

$$w_{random}\left( SL \right)=1,$$

$$w_{size selection}\left( SL \right)=\left\{ \begin{aligned} 1, (SL<s_{1}) \\ \frac{(s_{2}-SL)}{(s_{2}-s_{1})}, (s_{1}\leq SL\leq s_{2}) \\ 0, \left( SL>s_{2} \right), \end{aligned} \right.$$

$$w_{growth selection}\left( SL,age \right)=\left\{ \begin{aligned} 1, (st(SL,age)<g_{1}) \\ \frac{\left( g_{2}-st\left( SL,age \right) \right)}{\left( g_{2}-g_{1} \right)}, (g_{1}\leq st(SL,age)\leq g_{2}) \\ 0, \left( st\left( SL,age \right)>g_{2} \right), \end{aligned} \right.$$

where $st\left( SL,age \right)=\frac{SL-\bar{a}*age-\bar{b}}{\sqrt{\bar{c}*age}}$.

Note that s_1_, s_2_ and g_1_, g_2_ are the parameters that control the ranges of size and growth selections, respectively, and that the random selection model has no parameters. The probability function of SL at a given age for the consumed juveniles (*P_c_*(*SL*, *age*)) was defined as:

$$P_{c}\left( SL,age \right)=\frac{\bar{P_{o}}\left( SL,age \right)*w}{\int_{-\infty}^{\infty} \bar{P_{o}}\left( SL,age \right)*w dSL}$$

where *w* is one of *w_random_, w_size selection_*, and *w_growth selection_*, and likelihood *L_c_* was calculated as follows:

$$L_{c}=\prod_{i} P_{c}\left( {SL}_{i}, {age}_{i} \right).$$

Parameters that allow *L_c_* to reach its maximum (*L_cmax_*) were estimated for each model. Bootstrap likelihood ratio tests were performed with 2,000 iterations using the random predation model as the null hypothesis for the other two models. In addition, Akaike’s Information Criteria (AIC) was calculated as AIC = -2*log(*L_cmax_*) + 2*k, where k is the number of parameters (k = 0 for the random predation model and k = 2 for the size and growth selection models; the model with the lowest AIC was selected. These procedures were applied to the data of each study site, pooled data for each year, and pooled data for all years. However, study sites where significant selective rejection of the head may have occurred were excluded. These analyses were performed using a custom program written in Python 3.8.5 using libraries pandas 1.1.3^S6^, numpy 1.19.2^S7^, and scipy 1.5.2^S8^.

**S3. Mean abundance and biomass of the top 10 fish and cephalopod species during 2008–2010 in the southern ECS.**

**Figure S2**. Mean abundance (left) and biomass (right) of the top 10 fish and cephalopod species collected from the codend and cover net of the bottom trawl during 2008–2010 in the southern East China Sea south of 30°N from sites with stomach content data. Horizontal bars indicate standard deviation of the mean. Shaded and solid bars indicate *Trachurus japonicus* and *Uroteuthis edulis*, respectively. Open circles denote species-specific occurrence in the study area.

**Table S1**. Body size, wet weight, and digestion states of juvenile *Trachurus japonicus* estimated using otoliths in stomachs of *Uroteuthis edulis* in the southern East China Sea.

| Collection | | *U. edulis* | | Stomach WW (mg) | *T. japonicus* | | | Others | Digestion state | | Time at predation |
| --- | --- | --- | --- | --- | --- | --- | --- | --- | --- | --- | --- |
| Year | Site | ML (mm) | WW (g) |  | N | SL (mm) | Total WW (g) | N | Rate (%) | Duration (min.) |  |
| 2008 | St. 04 | 127 | 74.0 | - | 7 | 28.4 - 48.7 | 6.1 | 0 | - | - | - |
|  |  | 134 | 86.8 | - | 3 | 20.0 - 38.0 | 1.4 | 0 | - | - | - |
|  |  | 112 | 54.0 | - | 1 | 43.4 | 1.2 | 0 | - | - | - |
|  |  | 125 | 66.0 | - | 1 | 40.2 | 1.0 | 0 | - | - | - |
| 2009 | St. 06 | 160 | 119.9 | 648 | 1 | 57.0 | 2.5 | 0 | 25.9 | 191 | 3:42 |
|  |  | 110 | 50.5 | 355 | 1 | 49.7 | 1.6 | 0 | 22.2 | 212 | 3:21 |
|  |  | 108 | 45.1 | 308 | 1 | 50.0 | 1.6 | 0 | 19.3 | 228 | 3:04 |
|  |  | 104 | 44.1 | 688 | 1 | 60.4 | 3.0 | 0 | 22.9 | 208 | 3:25 |
|  |  | 87 | 25.0 | 254 | 1 | 47.5 | 1.4 | 0 | 18.1 | 235 | 2:58 |
|  | St. 15 | 138 | 81.3 | 879 | 1 | 53.3 | 2.0 | 0 | 44.0 | 88 | 4:55 |
|  |  | 147 | 104.5 | 2165 | 5 | 37.4 - 48.7 | 5.5 | 0 | 39.4 | 114 | 4:29 |
|  |  | 104 | 39.1 | 413 | 1 | 44.7 | 1.1 | 0 | 37.5 | 125 | 4:19 |
|  | St. 18 | 75 | 18.6 | 196 | 1 | 44.4 | 1.1 | 0 | 17.8 | 237 | 5:32 |
|  |  | 81 | 21.2 | 189 | 1 | 40.1 | 0.8 | 0 | 23.6 | 204 | 6:05 |
|  |  | 58 | 9.5 | 178 | 1 | 40.7 | 0.8 | 0 | 22.3 | 211 | 5:57 |
|  | St. 19 | 108 | 46.1 | 271 | 1 | 42.1 | 0.9 | 0 | 30.1 | 167 | 3:37 |
|  |  | 122 | 67.4 | 1819 | 3 | 40.3 - 55.7 | 4.3 | 0 | 42.3 | 97 | 4:46 |
|  |  | 119 | 69.1 | 656 | 2 | 37.9・45.1 | 1.9 | 0 | 34.5 | 142 | 4:02 |
|  |  | 160 | 122.1 | 2478 | 1 | 46.9 | 1.4 | 1* | - | - | - |
|  |  | 108 | 47.0 | 348 | 2 | 30.8・38.4 | 1.1 | 0 | 31.6 | 158 | 3:45 |
|  |  | 74 | 19.0 | 325 | 1 | 43.5 | 1.1 | 0 | 29.5 | 170 | 3:34 |
| 2010 | St. 14 | 120 | 65.0 | 468 | 2 | 20.9・34.1 | 0.6 | 3* | - | - | - |
|  |  | 133 | 79.1 | 1982 | 4 | 29.3 - 41.3 | 2.8 | 0 | 70.8** | - | - |
|  |  | 125 | 75.0 | 1139 | 1 | 36.7 | 0.7 | 1* | - | - | - |
|  | St. 19 | 140 | 92.5 | 83 | 1 | 73.8 | 6.2 | 0 | 1.3 | 330 | 0:46 |
|  |  | 114 | 62.2 | 469 | 1 | 74.9 | 6.5 | 0 | 7.2 | 297 | 1:20 |
|  |  | 107 | 48.4 | 777 | 1 | 49.0*** | 1.7 | 0 | 46.8 | 72 | 5:05 |
|  |  | 111 | 59.3 | 996 | 1 | 61.2 | 3.4 | 0 | 29.4 | 171 | 3:26 |
|  |  | 114 | 56.1 | 969 | 1 | 65.6 | 4.2 | 0 | 22.8 | 208 | 2:48 |
|  |  | 117 | 63.8 | 1414 | 2 | 54.7・66.7 | 6.8 | 0 | 20.8 | 220 | 2:37 |
|  |  | 115 | 56.4 | 828 | 1 | 67.1 | 4.6 | 0 | 18.2 | 235 | 2:22 |
|  |  | 76 | 22.0 | 573 | 1 | 60.1 | 3.2 | 0 | 17.9 | 236 | 2:20 |
|  |  | 115 | 55.8 | 1127 | 1 | 66.5 | 4.4 | 0 | 25.4 | 193 | 3:03 |
|  |  | 103 | 40.3 | 617 | 1 | 61.9 | 3.5 | 0 | 17.6 | 238 | 2:18 |

*Squids digesting other fish species (3 out of 34 individuals) were excluded from the analysis for digestion state and time at predation.

**As digestion ratio > 59.4 % was not available for the evacuation equation (Yasui and Sakurai 2005), the subsequent analysis for digestion duration and time at predation was not estimated in this case.

***As no data on otolith growth increments was available, this individual was excluded from the subsequent analysis.

**References**

S1. Takahashi, M., Sassa, C., Nishiuchi, K. & Tsukamoto, Y. Interannual variations in rates of larval growth and development of jack mackerel (*Trachurus japonicus*) in the East China Sea: implications for juvenile survival. *Can. J. Fish. Aquat. Sci.* **73**, 155–162 (2016).

S2. Rodhouse, P. G. & Nigmatullin, C. M. Role as consumers. *Phil. Trans. R. Soc. B* **351**, 1003-1022 (1996).

S3. Lipiński, M. R. Food and feeding of *Loligo vulgaris reynaudii* from St. Francis Bay, South Africa. *S. Afr. J. Mar. Sci.* **5**, 557-564 (1987).

S4. Tokai, T., Shiode, D., Sakai, T. & Yoda, M. Codend selectivity in the East China Sea of a trawl net with the legal minimum mesh size. *Fish. Sci.* **85**, 19–32 (2019).

S5. Kishida, M. *et al*. Ecomorphological dimorphism of juvenile *Trachurus japonicus* in Wakasa Bay, Japan. *Environ. Biol. Fish.* **90**, 301–315 (2011).

S6. McKinney, W. Data structures for statistical computing in python in *Proc. of the 9th Python in Science Conf.* http://conference.scipy.org/proceedings/scipy2010/ (eds. Van der Walt, S. & Millman, J.) 56–61 (2010).

S7. Harris, C. R. *et al*. Array programming with NumPy. *Nature* **585**, 357–362 (2020).

S8. Virtanen, P. *et al*. SciPy 1.0: fundamental algorithms for scientific computing in Python. *Nat. Methods* **17**, 261–272 (2020).
